# An Experimental Performance Study of a Catalytic Membrane Reactor for Ethanol Steam Reforming over a Metal Honeycomb Catalyst

**DOI:** 10.3390/membranes11100790

**Published:** 2021-10-18

**Authors:** Nikita Eremeev, Alexey Krasnov, Yuliya Bespalko, Ludmilla Bobrova, Oleg Smorygo, Vladislav Sadykov

**Affiliations:** 1 Federal Research Center Boreskov Institute of Catalysis SB RAS, 630090 Novosibirsk, Russia; intoleadensky@gmail.com (A.K.); bespalko@catalysis.ru (Y.B.); lbobrova@catalysis.ru (L.B.); sadykov@catalysis.ru (V.S.); 2 O.V. Roman Powder Metallurgy Institute, 220005 Minsk, Belarus; smorygo@tut.by

**Keywords:** ethanol steam reforming, asymmetric supported membrane, hydrogen separation, membrane reactor modeling

## Abstract

The present study deals with the combination of ethanol steam reforming over a monolithic catalyst and hydrogen separation by membrane in a lab-scale catalytic membrane reactor (CMR). The catalyst was comprised of honeycomb thin-walled Fechralloy substrate loaded with Ni + Ru/Pr_0.35_Ce_0.35_Zr_0.35_O_2_ active component. The asymmetric supported membrane consisted of a thin Ni-Cu alloy–Nd tungstate nanocomposite dense permselective layer deposited on a hierarchically structured asymmetric support. It has been shown that the monolithic catalyst-assisted CMR is capable of increasing the driving potential for hydrogen permeation through the same membrane as compared with that of the packed bed catalyst by increasing the retentate hydrogen concentration. Important operating parameters responsible for the low carbon deposition rate as well as the amount of hydrogen produced from 1 mol of ethanol, such as the temperature range of 700–900 °C, the water/ethanol molar ratio of 4 in the feed, have been determined. Regarding the choice of the reagent concentration (ethanol and steam in Ar), its magnitude may directly interfere with the effectiveness of the reaction-separation process in the CMR.

## 1. Introduction

Integrated options for production of a hydrogen enriched gas and for hydrogen recovery by membrane separation in a single device give prospects to process miniaturization, continuous operation, and energy saving. Mass transport across membranes between two gas streams (retentate and permeate) may occur under induced driving forces. A wide range of driving forces for the transmembrane transport in terms of gradients in the chemical potential, the electrical potential, and the hydrostatic pressure, which could result in diffusion of individual molecules, a migration of ions, and a convection of mass, respectively, is framed in [1]. A variety of membrane separation processes are often classified according to their driving forces [2]. Catalytic membrane reactors are able to provide a high conversion of fuels with the advantage of producing a very pure hydrogen stream supply [3,4,5,6,7,8].

Performance of catalytic membrane reactors strongly depends on the magnitude of the driving force and on the permeability of a membrane. The driving force for hydrogen permeation across the membrane at the ambient pressure is a hydrogen partial pressure gradient at the sides of membrane. Additionally, both the catalyst and membrane need to be adapted to provide a low overall resistance to mass transport of hydrogen.

Previously, a packed bed membrane reactor for production of hydrogen through ethanol steam reforming has been successfully tested at ambient pressure and the temperature range of 700–900 °C at a laboratory scale [9,10,11,12,13]. The asymmetric membrane disk module consisted of a gas-tight nanocomposite functional coating (Ni + Cu/Nd_5.5_WO_11.25-δ_ mixed proton-electron conducting nanocomposites) deposited on a gas-permeable functionally graded foam substrate. The membrane was placed immediately downstream from a packed bed consisting of spherical catalyst particles of 1 mm diameter. The best results have been obtained at 900 °C and feed of ethanol/H_2_O mixture in Ar at steam-to-ethanol ratio of four. An overall hydrogen flux was achieved to be about 1.31 Nml cm^−2^ min^−1^. Numerical study of interrelated phenomena of catalytic reaction and permeation through the asymmetric membrane has shown a strong impact of the structural parameters of composite membranes on the reactor performance [14].

The mixture of water and ethanol/bioethanol is often used to produce the hydrogen-rich gas in catalytic membrane reactors. The highest amount of hydrogen is obtained by steam reforming of ethanol, so that theoretically 6 moles of hydrogen are formed per one mole of ethanol in the feed [15,16]:

(1) $${\mathrm{CH}}_{3}{\mathrm{CH}}_{2}\mathrm{OH}(g)+3H_{2}O(g)\to 6H_{2}(g)+2{\mathrm{CO}}_{2}(g)\;\;\;\;\;\Delta H_{298}^{o}=+157.09\;\;\mathrm{kJ}\cdot {\mathrm{mol}}^{-1}.$$

The ethanol steam reforming step-wise scheme can be presented as follows [7,10]:

C_2_H_5_OH + (OH)_S_ = (C_2_H_5_O)_S_ + H_2_O, (2)

(C_2_H_5_O)_S_ + H_2_O = (CH_3_COO)_S_ + 2 H_2_, (3)

(CH_3_COO)_S_ + (OH)_S_ = CH_3_CHO + 2 (O)_S_, (4)

(CH_3_COO)_S_ + (OH)_S_ = (CO_3_)_S_ + (   )_S_ + CH_4_, (5)

(CO_3_)_S_ + H_2_O = CO_2_ + 2 (OH)_S_, (6)

(CO_3_)_S_ + (   )_S_ = CO + 2 (O)_S_, (7)

C_2_H_5_OH = CH_4_ + H_2_ + CO, (8)

CH_4_ + H_2_O = CO + 3 H_2_, (9)

CO + H_2_O = CO_2_ + H_2_, (10)

CH_4_ + 2 H_2_O = CO_2_ + 4 H_2_, (11)

2 CO = CO_2_ + C. (12)

Ethanol molecules dissociate on catalyst active sites to form ethoxy species (Equation (2)) which transform into acetyl species (Equation (3)) and then acetaldehyde (Equation (4)) as well as carbon monoxide and dioxide after demethanation (Equations (5)–(7)). The scheme includes ethanol decomposition (Equation (8)), methane steam reforming (Equation (9)), water-gas shift reaction (Equation (10)), complex shift reaction (Equation (11)) and the Boudouard reaction (Equation (12)) as well [7,10].

The stoichiometric molar steam-to-ethanol ratio (S/E) for complete conversion of coreactants to carbon dioxide and hydrogen is 3 (Equation (1)). Higher ratios can also be used to produce more H_2_ due to positive kinetic effect of the excessive steam supplied to the reaction. According to the thermodynamic study performed by Sun et al. [17], for the reforming reaction with the H_2_, CO, CO_2_, and CH_4_ productions, at about 1000 K more than 5.1 mol of H_2_ (close to 90% of equilibrium yield) can be obtained at S/E > 8. The CO_2_ yield maximizes at about 900 K with S/E > 8. Yet, increasing the S/E molar ratio above 6 has been found to decrease the hydrogen recovery, and more energy is required for feed preparation [18,19].

The operating parameters of an integrated fuel-processing catalytic system are crucial to exploit the full potential of a membrane itself. Characteristics of a catalyst, such as its design and levels of intrinsic activity and selectivity sufficient to compete with membrane efficiency, are essential for creating a high hydrogen gradient all over the membrane module. Thus, packed beds consisting of particles (typically 1–10 mm) may offer significant mass transfer limitations, thus lowering the driving potential for the transmembrane permeation [20]. Catalyst localization and loading are also of importance in the performance of catalytic membrane reactors. A great role of the pore size and porosity of the catalytic layer on hydrogen production is demonstrated in [21]. A reaction zone located in the vicinity of the membrane will create hydrogen-rich environment in the membrane headspace, thus increasing the partial pressure gradient. Alternatively, a rather large distance between catalyst and membrane may lead to a decrease in H_2_ partial pressure toward the membrane surface. Among factors influencing the net driving force of hydrogen transport, the fluid dynamics being highly dependent on the chosen experimental reactor configuration and operating conditions is very important [22,23].

It is known that structural catalysts (honeycomb monoliths as well as microchannel plates) with a thin catalytically active layer of about 0.01 mm thickness washcoated over metallic substrates provide both a low pressure drop and a low pore diffusion resistance, hence, a high activity. The mini-channels with characteristic diameters between 400 μm and about 1 mm have large surface to volume ratios (catalytically active surface area per the catalyst unit volume), which results in superior transfer properties and, consequently, offers a compact and modular solution for the devices. The excellent thermal conductivity of metallic monoliths provides more uniform temperature profiles along the catalysts length/diameters. Such benefits as their potential to intensify processes by enhancing heat and mass transfer or by more precisely controlling contact times, which may also improve the process efficiency, arise from a specific flow regime appearing in small channels—nearly plug flow behavior [24,25].

The objective of this study was to employ a metallic honeycomb catalyst in a lab-scale catalytic membrane reactor in order to produce hydrogen through ethanol steam reforming. Water and ethanol reacted over the monolithic catalyst yielding a hydrogen-rich gas. A disc-shaped asymmetric supported membrane was placed immediately downstream from the catalyst to extract the hydrogen from the product gas. The asymmetric supported membrane consisted of a thin Ni-Cu alloy–Nd tungstate nanocomposite dense permselective layer deposited on a Ni-Al hierarchically structured asymmetric support with graded porous structure. The aim was to evaluate the experimental reactor performance (in terms of conversion and selectivity, the permeation rate of hydrogen through the membrane) at different operating conditions regarding temperature, flow rates of the fuel ethanol-water mixture, and different water/ethanol molar ratios. As a result, optimal conditions of carrying out the reforming reaction over the monolith simultaneously with the hydrogen separation to achieve the efficient hydrogen production in the experimental CMR were established.

## 2. Materials and Methods

### 2.1. Synthesis of Materials

The Nd_5.5_WO_11.25-δ_ (NW) powder synthesized by the mechanical activation as described elsewhere [26] was characterized as a single-phase defect fluorite [7,26]. The NiCu alloy was synthesized by modified Pechini route using a fluidized bed reactor for subsequent thermal treatment of products of polymeric precursors decomposition in Ar + H_2_ streams. The obtained nanoparticles were put into isopropanol to avoid oxidation. The NiCu (30 wt.%)/Nd_5.5_WO_11.25-δ_ nanocomposite was prepared by ultrasonic dispersion of NiCu alloy and NW powder suspension in isopropanol with addition of polyvinyl butyral [7].

A metal substrate was formed by stacking flat and corrugated foil (Kanthal FeCrAl alloy) bands (each approximately 120 µm in thickness) and winding them into an Arkhimed spiral. The corrugated foil had a wave height of 0.7 mm at a pitch of 2.5 mm, so the entire cylindrical honeycomb substrate with outer diameter of 24 mm had 12 layers of flat and corrugated foils in the cross section. To prepare structured catalyst, first γ-Al_2_O_3_ (10 wt.%) was supported by washcoating, then an active component comprised of 10 wt.% Pr_0.35_Ce_0.35_Zr_0.35_O_2_ + 5 wt.% Ni + 1 wt.% Ru was supported by wetness impregnation as described elsewhere [27,28].

**Figure 1:**
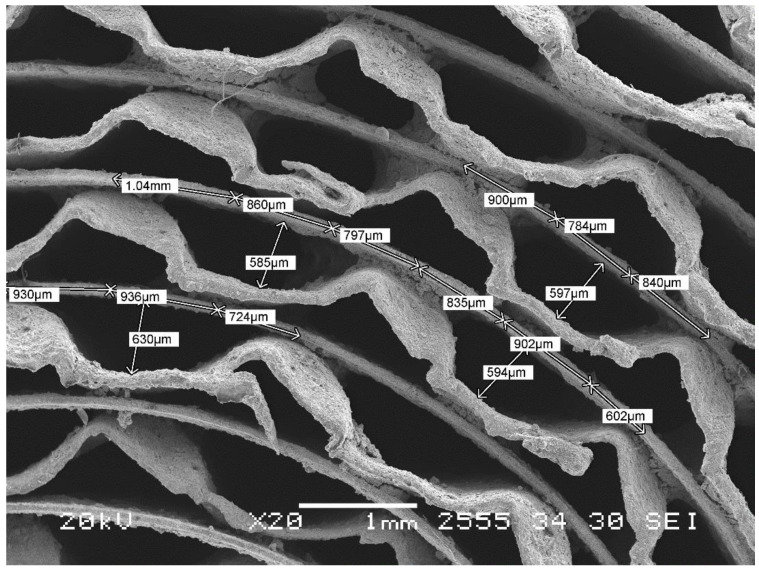
SEM image of the honeycomb catalytic monolith.

The performance of the catalytic honeycomb monolith is to a large extent determined by its structure, morphology, and porosity. Morphological evaluations of the monolith were made with a help of scanning electron microscopy (SEM) using a Jeol GSMT-300 instrument (Tokyo, Japan). A micrograph in Figure 1 allows us to quantify features related to key transport properties of the catalyst. The structural features of honeycomb monoliths are usually characterized by their geometric parameters such as specific surface area and porosity, which can be calculated from the channel dimensions and wall thickness. Thus, the geometric porosity was calculated using estimated cross areas, a geometric one, and the channel walls upon which the catalyst was deposited. Additionally, the influence of the sinusoidal corrugation was taken into account at calculation of the cross-section area of the channel walls. The equivalent/hydraulic channel diameter was calculated from Equation (13):

(13) $$D_{e}=\frac{4\;(Porosity)}{Specific\;geometric\;surface\;area}\cdot$$

Characteristics of the honeycomb catalyst are listed in Table 1.

### 2.2. Membrane Preparation

The asymmetric supported membrane consisted of a thin Ni-Cu alloy–Nd tungstate nanocomposite dense permselective layer with thickness of 0.1 mm deposited by vacuum slip casting on a Ni-Al hierarchically structured asymmetric foam substrate and sintered in Ar at temperatures up to 1100 °C [7,11,29,30]. The procedure was repeated until reaching necessary gas-leak tightness.

### 2.3. Evaluation of the Catalytic Membrane Reactor Performance

**Figure 2:**
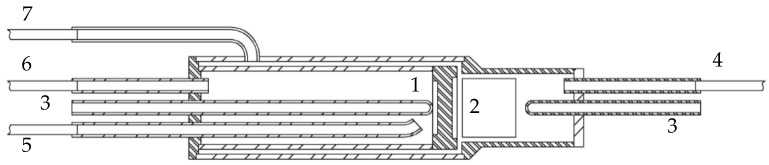
Scheme of catalytic reactor for ethanol steam reforming: 1—asymmetric supported hydrogen separation membrane, 2—honeycomb catalyst, 3—thermocouples pockets, 4—tube for feed gas mixture feeding, 5—purge (sweep) gas feeding tube, 6—permeate outlet tube, 7—bypass tube.

To integrate the fuel processing over honeycomb catalyst simultaneously with the hydrogen separation by using a disk-shaped membrane in the same unit, a lab-scale CMR was designed (Figure 2). A multi-tubular membrane reactor configuration of all-welded metal construction was applied [31]. The disk-shaped membrane of 5.65 mm thickness and 26 mm diameter was installed between the two concentric tubular chambers. The inlet tube, the feed-side compartment (high pH_2_), had a diameter of 28 mm and a length of 50 mm. The outlet chamber consisted of two concentric tubes: the inner one, the sweep-side compartment (low pH_2_), had a diameter of 34 mm and a length of 86 mm. The outer tube had a diameter of 38 mm, which increased the axial distance from the honeycomb catalyst to the membrane surface up to 4 mm. The side of the membrane also served as a distributor and a mixer for the product stream to promote uniform fluid velocity distributions. Increasing the diameter of the outer tube in the vicinity of the membrane allows for the deceleration of the fluid atop the membrane surface. Such deceleration zone favors a good contact between the exit flow and membrane surface.

The whole reactor system was then placed into a well-insulated cylindrical furnace in order to heat up the membrane and the feed gases to the required operating temperature. The temperature distribution was controlled by thermocouples to maintain the set reactor temperature. The feed mixture was provided by the bubbler feed system. A feed gas (EtOH + H_2_O + Ar) was fed into the fuel side compartment through the tube (4). A space above the honeycomb catalyst was filled with a layer of 3 mm of quartz balls, in order to ensure an even flow through the catalyst. Clearance between catalytic monolith and reactor wall was filled with compacted insulation material (mineral wool). A retentate gas was removed through a bypass passage (7). Argon was used as an external permeate-side sweep gas to obtain the partial pressure driving force required for hydrogen permeation at the ambient pressure. Argon was fed into the sweep-side compartment via the tube (5). Permeated hydrogen in sweep gas exits the permeate zone through the tube (6).

The concentrations of individual gas components H_2_, CO, CH_4_, and CO_2_ in retentate and permeate streams were continuously analyzed by a gas analyzer TEST-1 system (Bonair, Russia) equipped with IR absorbance, electrochemical, and polarographic sensors. The amount of H_2_O in the reactor effluent was not analyzed but estimated by analysis of the total molar balance. Analysis of liquid products collected at the end of each experiment at reactor outlet indicated that full ethanol conversion has been achieved in studied range of experimental parameters.

The experiments were carried out with constant flow rates of 5 Nl h^−1^ for the feed gas (ethanol and water mixture in argon) and of 10 Nl h^−1^ for the Ar sweeping gas. The concentrations of both ethanol and water were systematically varied from 3 to 30 vol.% and from 13 to 80 vol.%, respectively. The operating temperatures were in the range of 500–900 °C. To prevent both the reactor coking and rapid catalyst deactivation the regeneration procedure was done before each experiment by heating up the reactor to 700 °C under flow of steam.

CO_2_ and CH_4_ yield was calculated as follows:

(14) $$i\;\mathrm{yield}=\frac{i\left(\mathrm{mol}\;\;s^{-1}\right)}{2\cdot \mathrm{ethanol}\left(\mathrm{mol}\;\;s^{-1}\right)},$$

where *i* = CO_2_ or CH_4_. Syngas yield was calculated as follows:

(15) $$\mathrm{Syngas}\;\mathrm{yield}=\frac{\left(H_{2}\;\mathrm{permeated}+H_{2}\;\mathrm{retentate}\right)\left(\mathrm{mol}\;s^{-1}\right)}{6\cdot \mathrm{ethanol}\left(\mathrm{mol}\;\;s^{-1}\right)}+\frac{\mathrm{CO}\left(\mathrm{mol}\;s^{-1}\right)}{2\cdot \mathrm{ethanol}\left(\mathrm{mol}\;s^{-1}\right)}.$$

Carbon yield was estimated as follows:

(16) $$\mathrm{Carbon}\;\mathrm{yield}=\frac{\left(\mathrm{CO}+CO_{2}+{\mathrm{CH}}_{4}\right)\left(\mathrm{mol}\;s^{-1}\right)}{2\cdot \mathrm{ethanol}\left(\mathrm{mol}\;s^{-1}\right)}.$$

The effective activation energy was calculated according to Sievert’s law for hydrogen permeation flux, $J_{H_{2}}$ (Nml H_2_ cm^−2^ min^−1^), and Sievert’s permeance, $Pe_{H_{2}}$ (mol m^−2^ s^−1^ Pa^−0.5^) [14]. The hydrogen permeation flux obeys Sievert’s law:

(17) $$J_{H_{2}}=Pe_{H_{2}}\left(\sqrt{P_{H_{2},dm}}-\sqrt{P_{H_{2},pl}}\right),$$

where $P_{H_{2},dm}$ and $P_{H_{2},pl}$ are the hydrogen partial pressures on opposite sides of the dense layer. The permeance $Pe_{H_{2}}$ follows an Arrhenius-like equation:

(18) $$Pe_{H_{2}}=\frac{\Theta }{\delta }\exp\left(-\frac{E_{a}}{RT}\right),$$

where *δ* is the dense layer thickness, Θ is the permeation constant, and *E_a_* is the effective energy of hydrogen permeation. Since the dense layer thickness remains constant, in order to simplify the calculations, the estimation of *E_a_* was carried out in Arrhenius-like coordinates:

(19) $$\left(Pe_{H_{2}}\times \delta \right)\left(\mathrm{arb}.\mathrm{un}.\right)-\frac{1000}{T}\left(K^{-1}\right).$$

The overall efficiency of the integrated reaction-separation process in the lab-scale CMR at different operating conditions was also analyzed in terms of hydrogen recovery and yield of hydrogen. A hydrogen recovery factor was calculated as a molar ratio of hydrogen permeated through the membrane to hydrogen produced from the ethanol steam reforming reaction in the feed-side:

(20) $$\mathrm{Recovery}\;\;\;H_{2}=\frac{H_{2}\;\;\mathrm{permeated}\left(\mathrm{mol}\;s^{-1}\right)}{\left(H_{2}\;\mathrm{permeated}+H_{2}\;\mathrm{retentate}\right)\left(\mathrm{mol}\;s^{-1}\right)}\cdot 100\%.$$

As regards the evaluation of the efficiency of catalytic process in the CMR, the percent yield is calculated as the extent to which the reaction theoretical yield (six moles hydrogen per mole of reacted ethanol, once the ethanol steam reforming reaction is complete) is achieved:

(21) $$\mathrm{Yield}\;\;H_{2}=\frac{\left(H_{2}\;\mathrm{permeated}+H_{2}\;\mathrm{retentate}\right)\left(\mathrm{mol}\;s^{-1}\right)}{6\cdot \mathrm{ethanol}\left(\mathrm{mol}\;s^{-1}\right)}\cdot 100\%.$$

## 3. Results and Discussion

### 3.1. Performance of the Monolithic Catalyst for Steam Reforming of Ethanol for Hydrogen Generation

The experimental study of ethanol steam reforming over the honeycomb catalyst simultaneously with the hydrogen separation in the CMR showed a product distribution that changed significantly with temperature, concentration of ethanol, and water/ethanol molar ratios. In the next sections, features of catalytic process in the CMR are analyzed in detail with the goal of discovering the range of reaction conditions over which the reactor can operate efficiently.

#### 3.1.1. Carbon Balance

To elucidate the best conditions for the reaction-separation processes in the CMR, the performance of the monolithic catalyst was characterized at various temperatures, steam-to-ethanol molar ratios, and ethanol concentrations in the feed gas.

One of the most important aspects of fuel transformations is to find an “optimum operation window” and define a coke regeneration strategy to attain the reproducible reactor behavior. In the process of hydrogen production from ethanol, deactivation of the catalyst is generally cased by sintering of the metal particles and/or carbon deposition resulting from transformation of coke precursors (ethylene, acetaldehyde, acetone, acetic acid, etc.) [32,33,34,35,36,37,38,39,40,41,42,43,44]. In this work, the potential for carbonaceous compounds formation was examined by calculating carbon balance over the reactor taking into account only CO, CO_2_, and CH_4_ products, not so active in carbon deposition, and the content of which was continuously recorded by the gas analyzer. Periodic tests of condensate at the reactor exit have shown that ethanol is completely converted over the whole range of temperatures studied, whereas such byproducts as acetaldehyde, acetone, acetic acid, etc. were indeed observed. Since the main task of this article is to analyze membrane performance mainly depending upon the hydrogen content in the converted feed, these data are not shown here and will be presented elsewhere.

**Figure 3:**
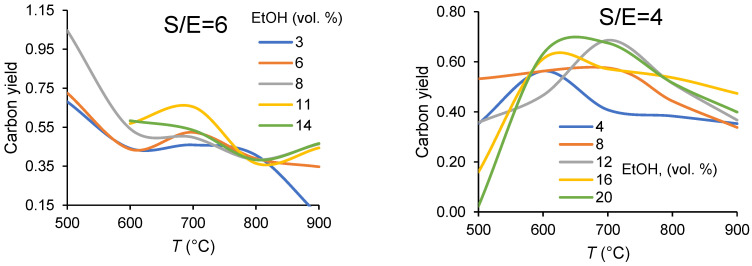

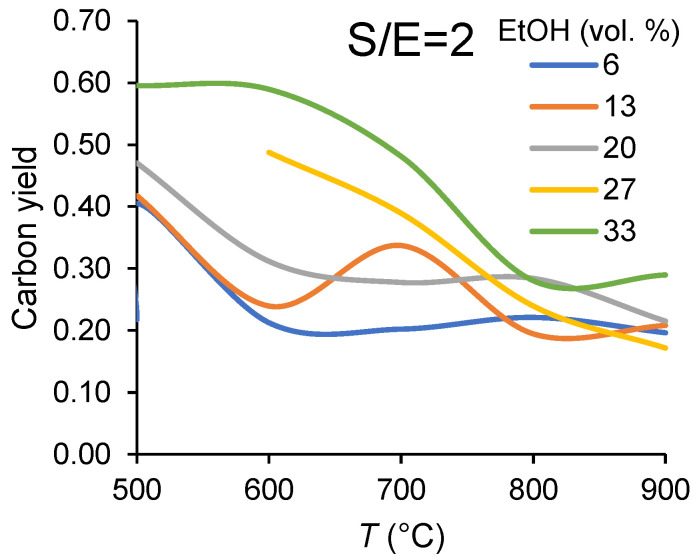
Dependencies of carbon yield in ethanol steam reforming in CMR at different ethanol concentrations (in vol.%) on temperature and the steam-to-ethanol molar ratios in the feed.

The results are shown in Figure 3. The values of carbon yield, which is the ratio of moles of carbon in the product gas to moles of carbon in ethanol, are set out in the 500–900 °C range for the steam-to ethanol molar ratios S/E = 2, 4, 6. The experiments were also conducted at different ethanol content. Since the flow rate of the feed (ethanol + water + argon) is constant (5NL h^−1^) in all experiments, the observed effect of the fuel concentrations in Ar also represents the effect of ethanol flow rates.

Since participation of water in reaction pathways apparently increases with temperature, high temperatures and steam-to-ethanol ratios promote hydrogen production. It can be seen from Figure 3 that operating parameters play a significant role in the carbon balance. The observed behavior of carbon yield as the temperature increases from 500 to 900 °C is rather intricate and seems to be dependent on the product distribution. Some intermediates and byproducts being formed in side reactions, such as acetaldehyde, ethylene, and methane, can be easily transformed into carbonaceous compounds. Thus, according to mass spectrometric studies of the gas phase in reaction conditions [35], acetaldehyde, hydrogen, and methane may exist irrespectively of the presence of water. Today, it is generally accepted that while low reaction temperatures favor formation of carbon through the Boudouard reaction or ethylene formation and polymerization, carbon formation through the decomposition of hydrocarbons is the main routes at higher temperatures [36,37]. It is evident that steam reforming reactions with their high production rate for hydrogen, which can be both a primary and a secondary product, require high temperatures, 700–900 °C. The lack of available intermediate products could result in smaller quantities of carbonaceous residues and the consequent lower deactivation rate at high temperatures. However, the results of the experiments give evidence of the high carbon deposition probabilities for all studied S/E ratios and even at high temperatures. Thus, carbon atoms fractions in compounds which could be involved in carbon formation are ~40–70% for S/E = 6, ~35–70% for S/E = 4 and ~20–50% and for S/E = 2 at 700 °C. Thus, Vicente et al. [38] has confined that the S/E molar ratio may have a low impact on the catalyst stability. After all, combinations of a catalyst and reaction conditions may influence greatly the formation of carbonaceous compounds. In any case, the phenomenon being observed requires further studies on the catalyst and reaction process.

**Figure 4:**
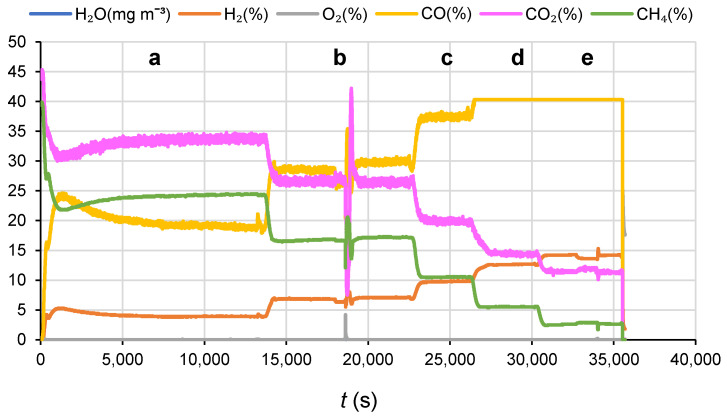
Concentration of ethanol steam reforming reaction products on retentate side of the membrane at different conditions: S/E = 2, ethanol feed concentration 27 vol.%, *T* = 500 °C (**a**), 600 °C (**b**), 700 °C (**c**), 800 °C (**d**), and 900 °C (**e**).

A noticeable effect of the ethanol concentration on the carbon yield can be also assigned to the effect of variation in product distribution with the space velocity that is the ratio of the flow rate of reactants to the catalyst volume. Indeed, since the flow rate of the feed (ethanol + water + argon) is constant (5 Nl h^−1^) in all experiments, the observed effect of fuel concentration represents also the effect of fuel flow rate. Increasing carbon imbalance under operating conditions favoring the increase in ethanol concentration, such as a low temperature, the high flow rate, high ethanol partial pressure in the feed, is explained by more probable transformation of ethanol into reaction products—coke precursors when its surface coverage is high [38]. Therefore, based on the effect of operating variables such as temperature, fuel composition (steam-to ethanol molar ratio), and concentrations of reactants, both the reactor operating regime and regeneration procedures have to be optimized to deal with this problem. Note, though, that in optimized conditions coke deposition was not observed on both membrane surface and monolithic catalyst, thus agreeing with the stable performance of the membrane reactor (Figure 4).

#### 3.1.2. Catalyst Performance in Terms of Product Distribution

A complex behavior of the ethanol reforming in product formation and distribution depending on the catalyst used and the reaction conditions applied have been observed by Zanchet et al. [39]. Despite very fast ethanol decomposition, resulting products can also be influenced by heat and mass transfer [40].

**Figure 5:**
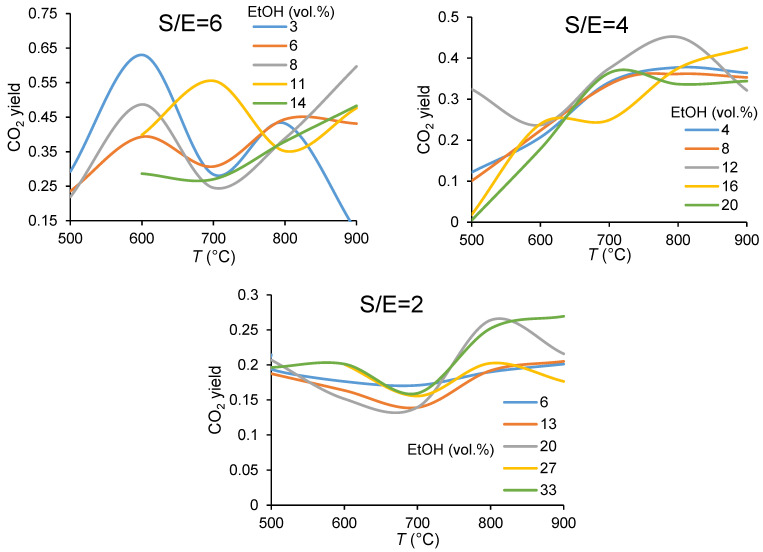
Temperature dependencies of carbon dioxide yield in ethanol steam reforming in CMR at the different ethanol concentrations (in vol.%) and the steam-to-ethanol molar ratios in the feed.

In the experiments analyzed here, the components detected as gas phase reaction products were hydrogen, carbon monoxide, carbon dioxide, and methane (Figures S1–S15, Supplementary Materials). A high carbon dioxide concentration in the feed gas is desirable from the point of view of preventing the formation of carbon by the Boudouard reaction (Equation (12)). Carbon dioxide yield in function of the operating temperature at different S/E molar ratio and degree of dilution by argon is shown in Figure 5. At low temperatures, CO_2_ formation mainly results from the oxidation of ethoxy species by the surface oxygen species and OH groups [7,41,42,43,44,45]. Evolutions of the CO_2_ yield as the temperature increases verify the involvement of the water molecules into intermediate reactions affecting the product distribution. It can be seen that, in general, the higher steam-to-carbon ratio leads to a higher conversion of ethanol to CO_2_. Carbon dioxide formation is also favored at a high concentration of ethanol. However, the main factor governing the reaction pathway to CO_2_ formation mostly happens to be the steam-to-ethanol molar ratio. Moreover, its intricate behavior versus the temperature at S/E = 6 gives evidence of water as a dominant factor in reaction pathways to its formation, possibly through the methane reforming which becomes more important, along with the reverse water gas shift reaction (Equation (10)), at higher temperatures.

Considering the effect of steam-to-ethanol ratio on CO_2_ content in the product gas, increasing the S/E ratio from 4 to 6 does not result in a significant increase of carbon dioxide content at high operating temperatures. Therefore, at high operating temperatures a reduction in the requirement of steam content fed to the reactor is also possible.

**Figure 6:**
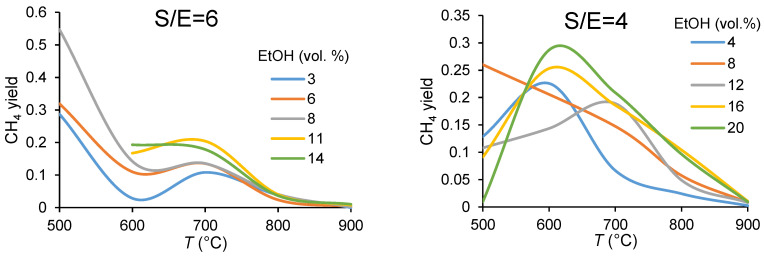

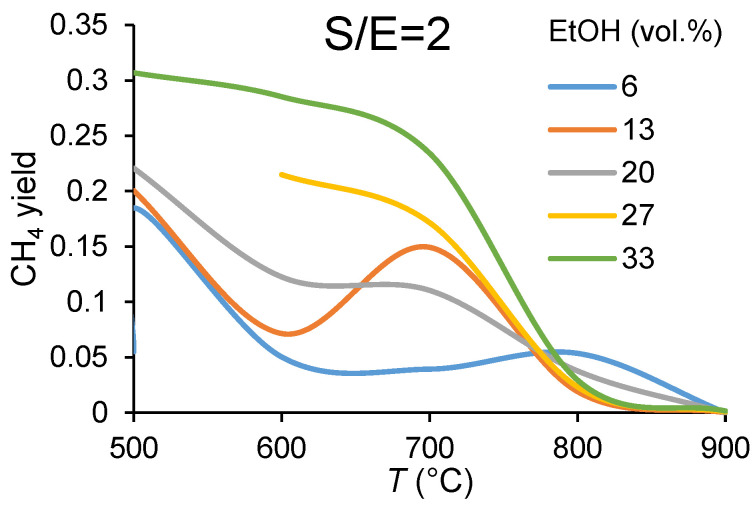
Temperature dependencies of methane yield in ethanol steam reforming in CMR at the different ethanol concentrations (in vol.%) and the steam-to-ethanol molar ratios in the feed.

As shown in Figure 6, for all S/E values methane yields are the highest at low temperatures. It is known that for the catalysts with a high oxygen mobility (that is the case), ethanol cracking, its oxidation to CO_2_ (Equation (11)) and methanation reaction (Equation (5), reverse reaction) that increases methane yield, occur at lower temperatures [7,46,47,48,49]. Indeed, the increase in temperature results in the sharp decrease of methane content by enhancing the reaction with steam (Equation (9)) in which the produced methane can be reformed to H_2_ and CO.

**Figure 7:**
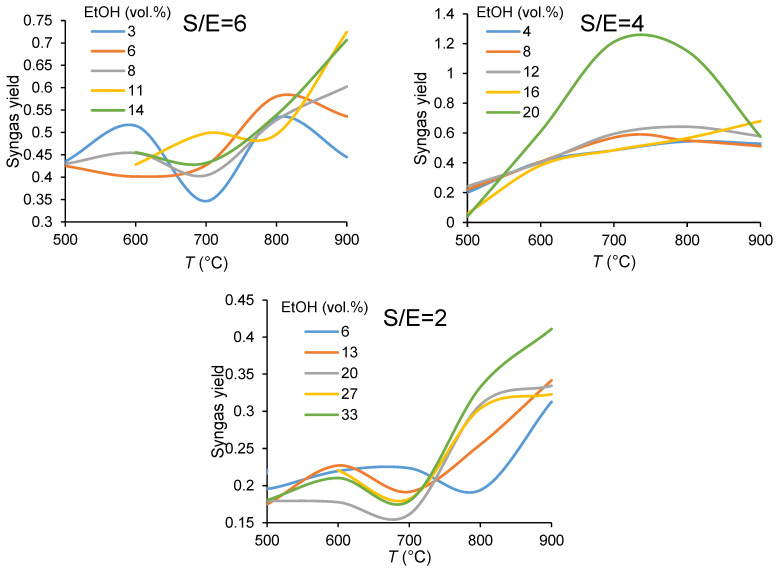
Temperature dependencies of syngas yield in ethanol steam reforming in CMR at different ethanol concentrations (in vol.%) and the steam-to-ethanol molar ratios in the feed.

Figure 7 demonstrates dependence of syngas yield on the process parameters. A strong temperature effect on the product distribution over the entire temperature range can be seen. The tendency to increase the amount of syngas with the operating temperature is obvious. However, dilution of the feed gas by Ar affects the syngas production quite ambiguously. It offers a better syngas yield at low temperatures, while maintaining the same concentrations; at higher temperatures a higher syngas yield was observed for feeds with a higher ethanol concentration.

**Figure 8:**
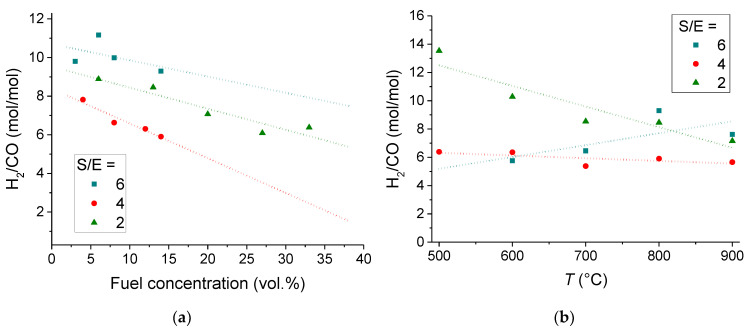
Hydrogen-to-carbon monoxide molar ratio in the syngas formed at the S/E = 6,4,2. (**a**) Effect of ethanol concentration at the operating temperature of 800 °C. (**b**) Effect of the temperature at the ethanol concentration in vol.%: of 14 (S/E = 6), 16 (S/E = 4), 13 (S/E = 2).

Of all the gas components present in the product gas, only hydrogen is able to permeate through the membrane. Concerning the driving force in the CMR, the syngas composition with a high hydrogen-to-carbon monoxide molar ratio is desirable as membrane reactor feed gas stream to meet the requirements for increasing the driving potential. Figure 8 presents the effect of the fuel concentration and temperature on the hydrogen-to carbon monoxide molar ratio in the syngas produced. A higher S/E ratio gives better selectivity to the production of hydrogen because both reactants (water and ethanol) contain hydrogen atoms and contribute to the hydrogen yield. The CO yield values do not exceed 0.2 in the temperature range from 500 °C to 700 °C. Secondary reactions, for instance Boudouard reaction (Equation (12)) or methane formation (Equations (5) and (8)), seem to be responsible for the low-level carbon monoxide yield. As the temperature is increased, both ethanol concentration and temperature enhance the trend toward the formation of carbon monoxide. At low ethanol concentrations the highest H_2_/CO ratios are obtained for S/E = 6.

**Figure 9:**
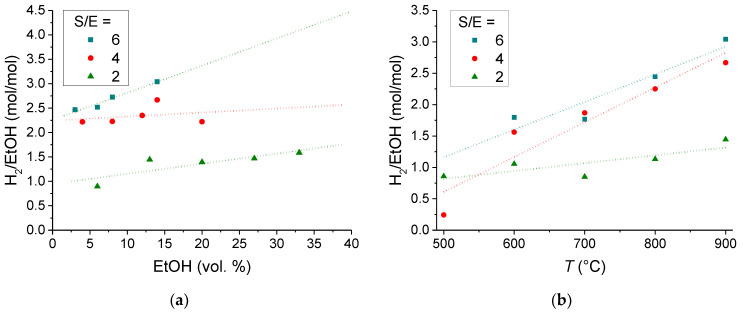
Effect of fuel concentration in Ar (**a**) at T = 900 °C and an operating temperature (**b**) on the amount of hydrogen produced from 1 mol of ethanol at the S/E = 6,4,2. (**b**) Data for the fuel concentration in vol.%: 14 (S/E = 6), 16 (S/E = 4), 13 (S/E = 2).

Figure 9 presents the influence of the fuel concentration and temperature on the amount of hydrogen produced from 1 mol of ethanol. It can be seen that for S/E = 4 and 2 ratios the ethanol concentration affects only slightly the amount of hydrogen produced from 1 mol of ethanol, of about 2.5 and 1.5 mol/mol (Figure 8a), respectively. The maximum yield of hydrogen is about 3 mol/mol for the S/E = 6 at the ethanol concentration of 14 vol %. The hydrogen yields, in terms of moles produced from 1 mol of ethanol, in the temperature range of 500–900 °C are given in Figure 8b. It can be seen that within the temperature range of 700–900 °C increasing the S/E ratio from 4 to 6 does not result in any notable increase of amount of hydrogen produced from 1 mol of ethanol.

Hence, detailed analysis of the monolithic catalyst performance in the ethanol steam reforming reaction carried out in this section demonstrated that main gas phase products formed were H_2_, CO, and CO_2_, with a small amount of CH_4_. This indicates a high activity and selectivity of catalyst the steam reforming of ethanol into. It is clear that to improve the overall efficiency of the process in a catalytic membrane reactor a lower steam to ethanol molar ratio should be used. With the goal of discovering the range of reaction conditions over which the catalyst can operate effectively, the water/ethanol molar ratio can be taken to be 4 at which the catalyst remained quite stable in the experiments.

### 3.2. Hydrogen Permeation Flux Analysis

Assessment of true potential of the dense NiCu (30 wt.%)–Nd_5.5_WO_11.25-δ_ permselective nanocomposite material in hydrogen permeation was carried out earlier from conductivity data [50,51]. Three distinct ranges of the proton conductivity in the temperature dependence in moist atmosphere of hydrogen, corresponding to different mechanisms of predominant proton conduction were revealed. The slope of the high-temperature plot (490–700 °C) results in an activation energy of ≈60 kJ mol^−1^ [14]. Generally, if the membrane permeation is limited by the surface reaction, the permeation flux has a linear dependence on the driving partial pressure force. Actual fluid dynamics (i.e., fluid flow, transfer characteristics, chemical reactions, etc.) in the catalyst-membrane assembly may also considerably affect the permeability data.

Hydrogen flux or permeation rate was estimated as a difference of hydrogen flowrates in retentate and permeate sections, per unit area of the membrane.

**Figure 10:**
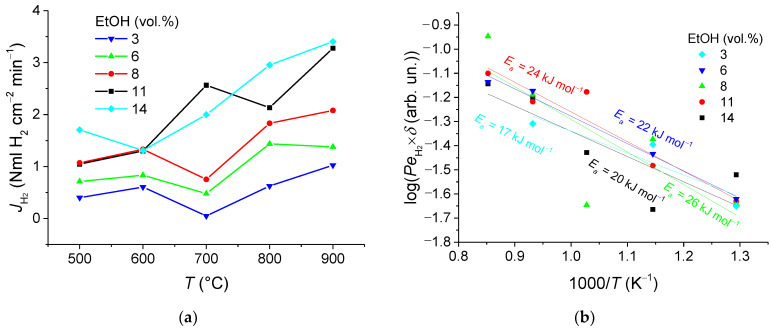
Hydrogen flux dependences on temperature (**a**) and corresponding Arrhenius plots (**b**) for S/E = 6 at the different fuel concentrations.

**Figure 11:**
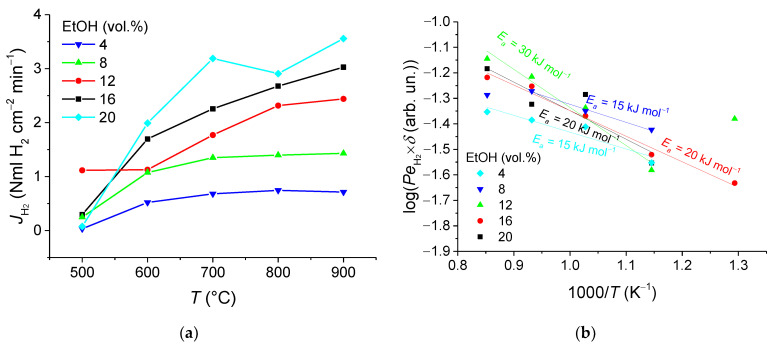
Hydrogen flux dependences on temperature (**a**) and corresponding Arrhenius plots (**b**) for S/E = 4 at different fuel concentrations.

**Figure 12:**
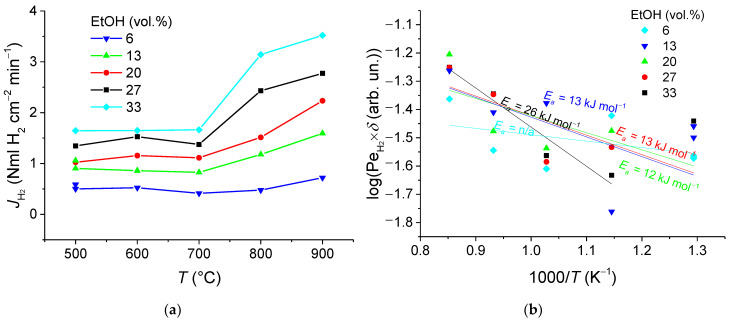
Hydrogen flux dependences on temperature (**a**) and corresponding Arrhenius plots (**b**) for S/E = 2 at the different fuel concentrations.

Figure 10a, Figure 11a, and Figure 12a show the H_2_ permeation as a function of temperature for various fuel contents in the feed gas with S/E = 6, 4, and 2, respectively. The findings give clear evidence that increases in the operating temperature and fuel concentration result in improved hydrogen permeation flux. Indeed, depending on conditions at the entry (feed side) and exit (sweep side) faces of the membrane, a concentration gradient establishing through the membrane gives rise to the membrane permeation. However, the maximum flux being observed at 900 °C, of 3.03–3.5 NmL H_2_ cm^−2^min^−1^, is nearly independent of the S/E ratio, while in the membrane reactor with a packed bed of the catalyst, this characteristic is calculated to be about 1.31 NmL cm^−2^ min^−1^ [11]. Thereby, this value is supposed to be a limit of the membrane permeation, when the diffusion flux through the membrane is no more governed by the driving partial pressure force, but rather by the diffusion through the membrane module. Thus, it was shown earlier [14] that the asymmetric support contributes up to 70% to the overall resistances across the membrane module.

Membrane permeability (Sievert’s permeance) was also assessed by using data on the permeated hydrogen flux versus temperature. The data reported were plotted in log scales as a function of reciprocal temperature (Figure 10b, Figure 11b, and Figure 12b), assuming that permeability of the asymmetric supported membrane of thickness *δ* depends on temperature according to Arrhenius law and Sieverts–Fick’s law [3,7,9,52]. The values for the effective activation energy were found to be in the range of 12–30 kJ mol^−1^, which are considerably lower than that of the process of hydrogen transfer through the NiCu alloy—Nd_5.5_WO_11.25-δ_ nanocomposite (60 kJ mol^−1^) [7,11,12]. This implies that the transport in support is a possible rate-controlling step of the hydrogen permeation through the 5.65 mm thick asymmetric supported membrane.

### 3.3. Reactor Performance Analysis

Efficiency of structured catalysts might be limited by the diffusion. In this context, the metallic monoliths could provide enhanced reaction rates due to a low pore diffusion resistance. Moreover, small channels diameter ensures a short radial diffusion time. The performance of the lab-scale CMR reactor with ethanol steam reforming over a metal honeycomb catalyst was also analyzed in terms of hydrogen recovery and yield.

**Figure 13:**
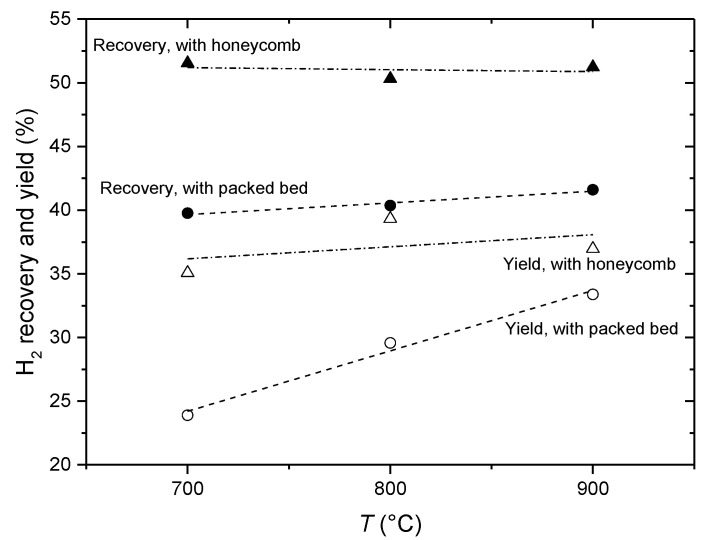
Comparison of the efficiency characteristics for CMRs with packed bed and honeycomb catalysts at the flow rates for the feed gas of 5 Nl h^−1^ and for the sweep gas of 10 Nl h^−1^. The feed gas: 4.0% of EtOH at S/E = 4 in Ar for honeycomb catalyst; 6.6% of EtOH at S/E = 5 in Ar for the fixed bed.

In order to quantify the impact of introducing a structural catalyst in the CMR reactor instead of a packed bed [7,14], hydrogen recovery and yield were evaluated through experiments being performed at the same flow rates, of 5 Nl h^−1^ for the feed gas (ethanol and water mixture in argon) and of 10 Nl h^−1^ for the Ar sweeping gas (Figure 13). These plots clearly indicate that the reactor operating with the catalytic monolith shows better performance in terms of both hydrogen recovery and the yield with respect to the packed bed catalyst. The monolithic honeycomb exhibits high and stable catalytic activity and selectivity in ethanol steam reforming within the range of operating temperatures. Thus, for the case with the packed bed catalyst, increasing the temperature from 700 °C up to 900 °C results in improving the yield of ethanol conversion to hydrogen by nearly 40%, while the yield for the honeycomb catalyst hardly changes (about 5%).

**Figure 14:**
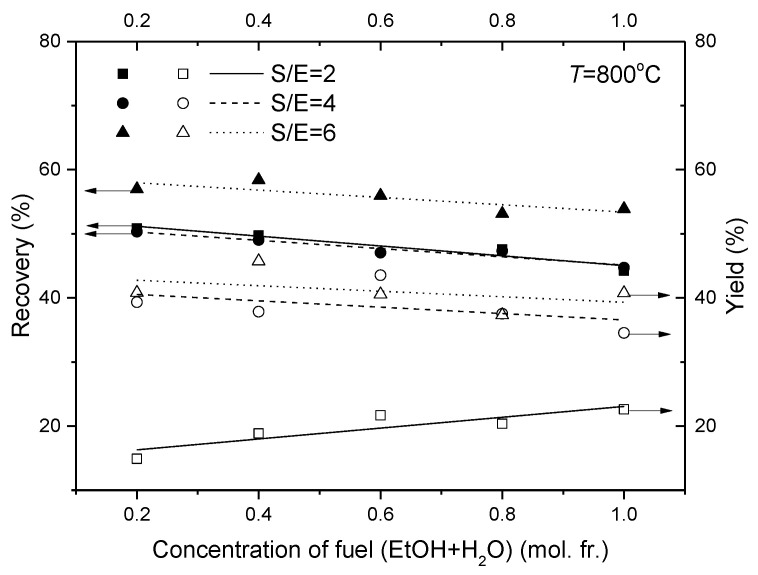
Effect of the concentration of fuel (EtOH + H_2_O) in Ar on the reactor efficiency characteristics. Operating temperature: 800 °C.

It is also extremely interesting to investigate the effect of both the S/E molar ratio and the fuel concentration towards the reforming reaction and hydrogen recovery. Hydrogen recovery is highly dependent on reactor operating conditions and permeation properties of a membrane. In Figure 14, the data for the hydrogen recovery decrease gradually as the fuel concentration increases. It has to be noted that the membrane shows better separating and recovering performance at the S/E = 6 mode due to a high amount of hydrogen produced from 1 mol of ethanol (see Figure 9a) compared to the operating conditions at the S/E = 4 and 2.

The influence of the fuel concentration on the hydrogen yield at S/E = 2 is opposite to those observed at S/E = 6 and 4. Indeed, providing sufficient H_2_O for the reaction contributes to efficient hydrogen production and, vice versa, less steam leads to a bigger contribution of ethanol.

**Figure 15:**
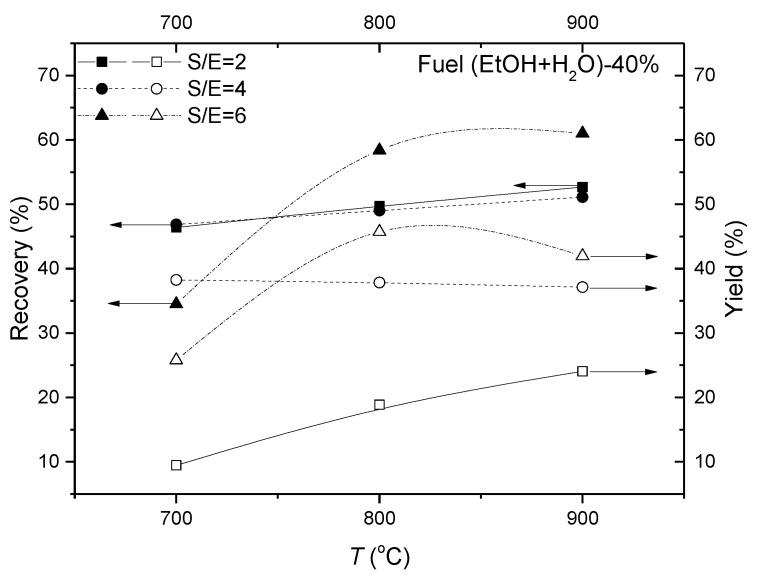
Effect of the operating temperature on the reactor efficiency characteristics for amount of the fuel mixture in Ar of 40%.

Effect of the operating temperature and steam-to carbon ratio in the fuel on the reactor efficiency characteristics can be evaluated from the plots in Figure 15. The impact of the temperature is greater at high S/E. Thus, the highest values of H_2_ recovery and yield are observed at S/E = 6 being in the ranges of 58–61 and 42–45%, respectively. The effectivity characteristics at S/E = 6 always exceed those at S/E = 4 mode at 800 and 900 °C, while at 700 °C high levels of both the hydrogen yield and its recovery were discovered only at the S/E = 4, to be of 37% and 47%, respectively.

Since a goal of the experimental study is to improve both the hydrogen-rich gas production and separation functions of the catalytic membrane reactor within entire operating temperature range, operation at the water/ethanol molar ratio of 4 in the fuel mixture is beneficial for ethanol conversion and hydrogen separation.

## 4. Conclusions

The CMR employing a metallic honeycomb catalyst for ethanol steam reforming reaction has been investigated extensively in this study with the goal to evaluate the experimental reactor performance at different operating conditions regarding temperature, fuel concentration, and different molar ratios between ethanol and water, in terms of efficient hydrogen production and permeation properties. In general, catalytic membrane reactor equipped with honeycomb catalyst outperforms the reactor with a packed-bed catalyst in terms of catalytic reaction performance and hydrogen permeability of the membrane. Intensification of the hydrogen flux has been obtained due to increasing hydrogen concentration in retentate gas. According to analysis of the experimental data presented above, the following conclusions can be made:

Increase in the fuel concentration (ethanol and steam) does not result in improving reactor performance characteristics;
The reactor operation in the temperature range of 700–900 °C with the water/ethanol molar ratio of 4 in the feed is quite sufficient in terms of ethanol transformation into syngas and CO_2_ as well as the amount of hydrogen produced from 1 mol of ethanol;
A high flux of ~3.5 Nml H_2_ cm^−2^min^−1^ can be provided at the operating temperatures due to a high mixed protonic-electronic conductivity of NiCu alloy–Nd_5.5_WO_11.25-δ_ nanocomposite as membrane material, while a possible rate-controlling step of the hydrogen permeation through the 5.65 mm thick asymmetric supported membrane is the transport in support.

## Figures and Tables

**Table 1:** Parameters of catalyst monolith.

Length	22	mm
Diameter	24	mm
Cross section area	452.16	mm^2^
Channel wall thickness	125	μm
Equivalent channel diameter	0.6924	mm
Porosity	0.58	
Specific geometric surface area	3355	m^−1^

## Data Availability

Not applicable.

## Supplementary Materials

The following are available online at https://www.mdpi.com/article/10.3390/membranes11100790/s1, Figure S1: Dependencies of ethanol steam reforming products concentrations (a), ethanol conversion and product selectivities (b) on temperature. Steam to ethanol ratio 6, ethanol inlet concentration 3 vol.%, Figure S2: Dependencies of ethanol steam reforming products concentrations (a), ethanol conversion and product selectivities (b) on temperature. Steam to ethanol ratio 6, ethanol inlet concentration 6 vol.%, Figure S3: Dependencies of ethanol steam reforming products concentrations (a), ethanol conversion and product selectivities (b) on temperature. Steam to ethanol ratio 6, ethanol inlet concentration 8 vol.%, Figure S4: Dependencies of ethanol steam reforming products concentrations (a), ethanol conversion and product selectivities (b) on temperature. Steam to ethanol ratio 6, ethanol inlet concentration 11 vol.%, Figure S5: Dependencies of ethanol steam reforming products concentrations (a), ethanol conversion and product selectivities (b) on temperature. Steam to ethanol ratio 6, ethanol inlet concentration 14 vol.%, Figure S6: Dependencies of ethanol steam reforming products concentrations (a), ethanol conversion and product selectivities (b) on temperature. Steam to ethanol ratio 4, ethanol inlet concentration 4 vol.%, Figure S7: Dependencies of ethanol steam reforming products concentrations (a), ethanol conversion and product selectivities (b) on temperature. Steam to ethanol ratio 4, ethanol inlet concentration 8 vol.%, Figure S8: Dependencies of ethanol steam reforming products concentrations (a), ethanol conversion and product selectivities (b) on temperature. Steam to ethanol ratio 4, ethanol inlet concentration 12 vol.%, Figure S9: Dependencies of ethanol steam reforming products concentrations (a), ethanol conversion and product selectivities (b) on temperature. Steam to ethanol ratio 4, ethanol inlet concentration 16 vol.%, Figure S10: Dependencies of ethanol steam reforming products concentrations (a), ethanol conversion and product selectivities (b) on temperature. Steam to ethanol ratio 4, ethanol inlet concentration 20 vol.%, Figure S11: Dependencies of ethanol steam reforming products concentrations (a), ethanol conversion and product selectivities (b) on temperature. Steam to ethanol ratio 2, ethanol inlet concentration 6 vol.%, Figure S12: Dependencies of ethanol steam reforming products concentrations (a), ethanol conversion and product selectivities (b) on temperature. Steam to ethanol ratio 2, ethanol inlet concentration 13 vol.%, Figure S13: Dependencies of ethanol steam reforming products concentrations (a), ethanol conversion and product selectivities (b) on temperature. Steam to ethanol ratio 2, ethanol inlet concentration 20 vol.%, Figure S14: Dependencies of ethanol steam reforming products concentrations (a), ethanol conversion and product selectivities (b) on temperature. Steam to ethanol ratio 2, ethanol inlet concentration 27 vol.%, Figure S15: Dependencies of ethanol steam reforming products concentrations (a), ethanol conversion and product selectivities (b) on temperature. Steam to ethanol ratio 2, ethanol inlet concentration 33 vol.%.

## Nomenclature

*D_e_*	the equivalent/hydraulic channel diameter (m);
*E_a_*	effective activation energy for permeation (kJ mol^−1^);
$J_{H_{2}}$	hydrogen permeate flux through the asymmetric membrane (mol m^−2^ s^−1^);
$Pe_{H_{2}}$	Sieverts permeance (mol m^−2^ s^−1^ Pa^−0.5^);
*p*H_2_	hydrogen partial pressure (Pa);
*S*/*E*	steam-to-ethanol molar ratio;
*S*(H_2_)	selectivity with respect to hydrogen;
*S*(CH_4_)	selectivity with respect to methane;
*S*(CO)	selectivity with respect to carbon monoxide;
*S*(CO_2_)	selectivity with respect to carbon dioxide;
*T*	operation temperature (K) or (°C);
*x*(EtOH)	ethanol conversion;
**Greek letters**	
δ	dense layer thickness (m);
Θ	permeation constant (mol m^−1^ s^−1^ Pa^−0.5^)
