# Supplementary material for: An Experimental Performance Study of a Catalytic Membrane Reactor for Ethanol Steam Reforming over a Metal Honeycomb Catalyst
Source: Membranes (Basel). 2021 Oct 18;11(10):790. doi: 10.3390/membranes11100790 (PMC8540183; doi:10.3390/membranes11100790)
Supplement: Supplementary file 1 [file membranes-11-00790-s001.zip › membranes-1415544-supplementary.pdf]

An experimental performance study of a catalytic membrane reactor for ethanol steam reforming over a metal honeycomb catalyst

Nikita Eremeev, Alexey Krasnov, Yuliya Bespalko, Ludmilla Bobrova, Oleg Smorygo and Vladislav Sadykov

Supplementary materials

***S/E* = 6**

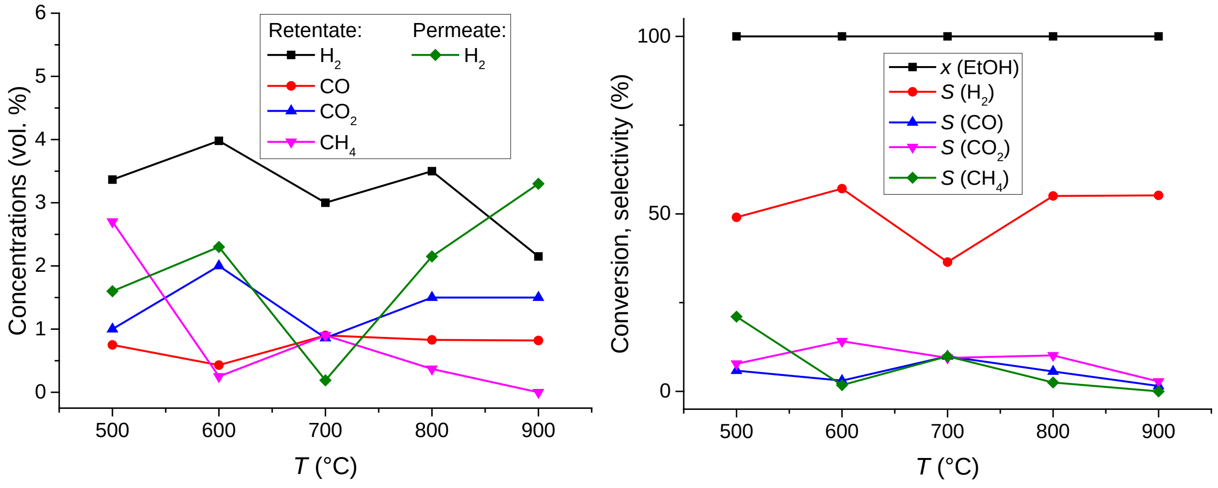

(**a**) (**b**)

Figure S1. Dependencies of ethanol steam reforming products concentrations (**a**), ethanol conversion and product selectivities (**b**) on temperature. Steam to ethanol ratio 6, ethanol inlet concentration 3 vol. %.

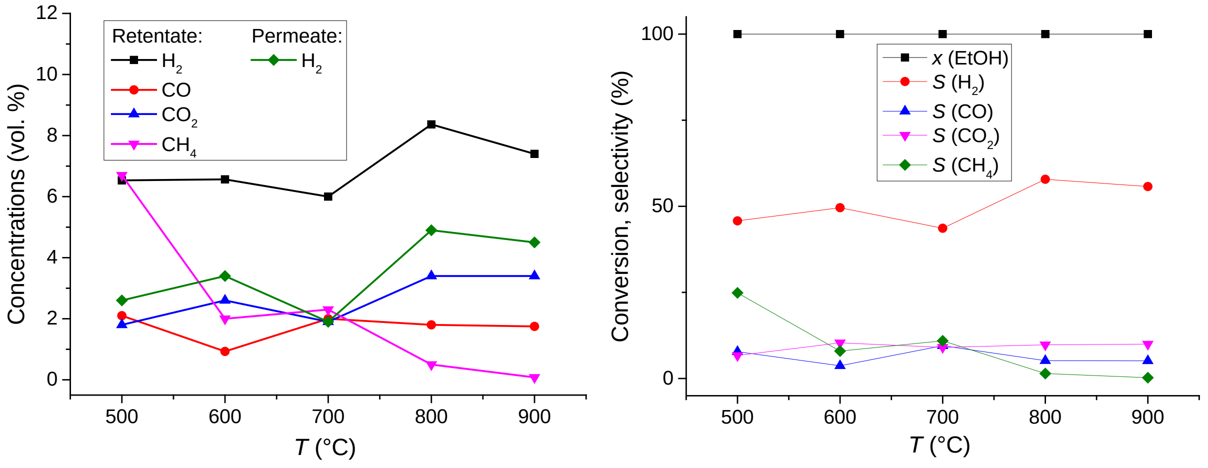

(**a**) (**b**)

Figure S2. Dependencies of ethanol steam reforming products concentrations (**a**), ethanol conversion and product selectivities (**b**) on temperature. Steam to ethanol ratio 6, ethanol inlet concentration 6 vol. %.

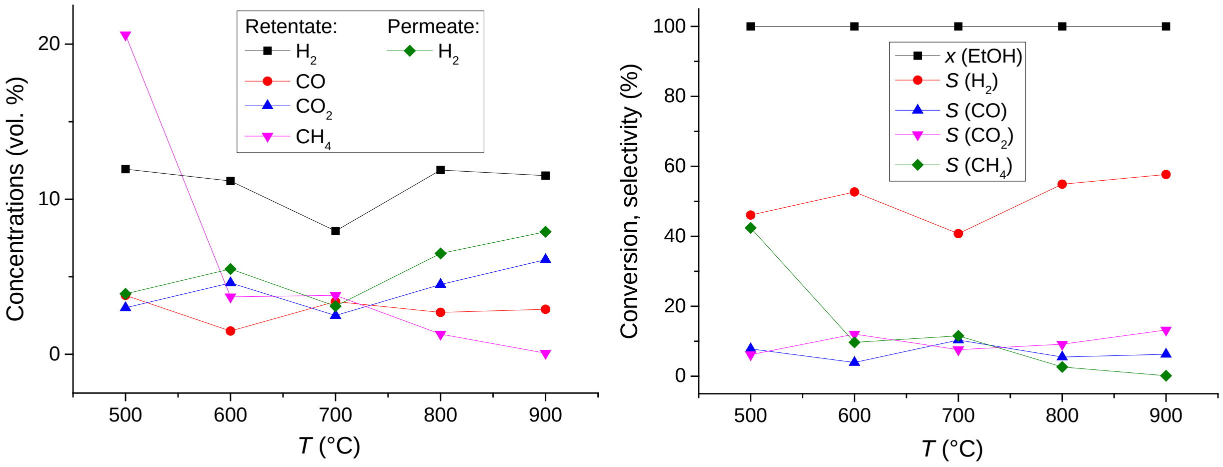

(**a**) (**b**)

Figure S3. Dependencies of ethanol steam reforming products concentrations (**a**), ethanol conversion and product selectivities (**b**) on temperature. Steam to ethanol ratio 6, ethanol inlet concentration 8 vol. %.

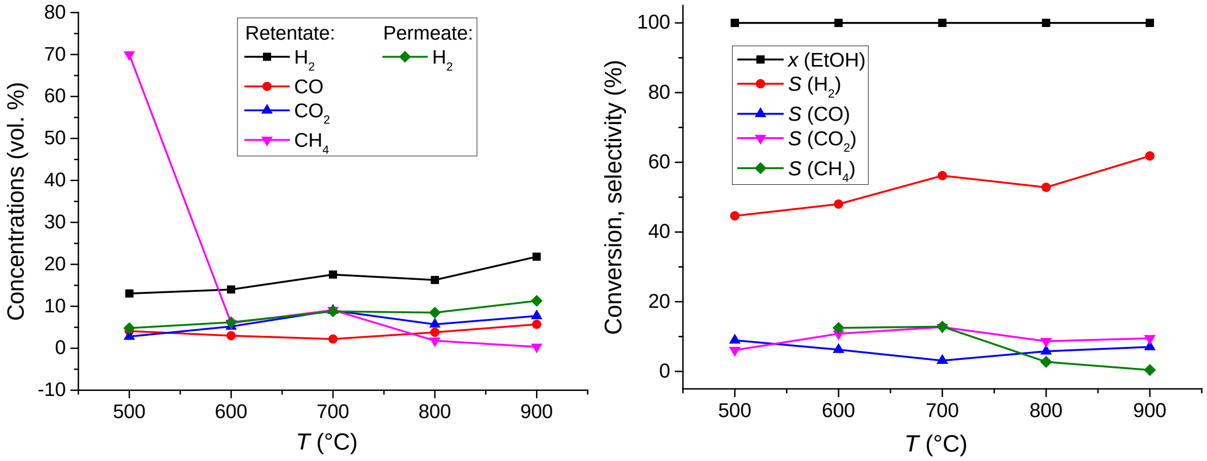

(**a**) (**b**)

Figure S4. Dependencies of ethanol steam reforming products concentrations (**a**), ethanol conversion and product selectivities (**b**) on temperature. Steam to ethanol ratio 6, ethanol inlet concentration 11 vol. %.

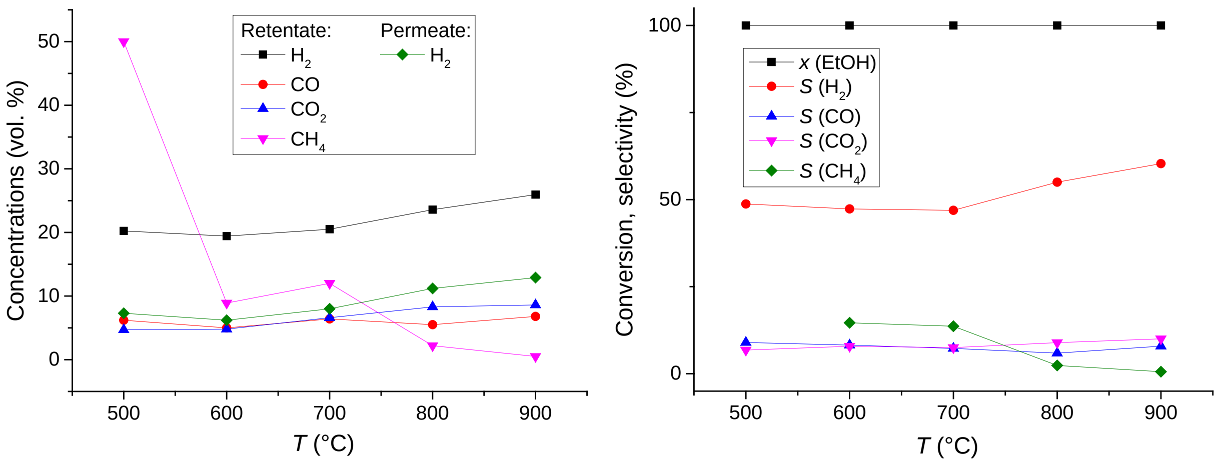

(**a**) (**b**)

Figure S5. Dependencies of ethanol steam reforming products concentrations (**a**), ethanol conversion and product selectivities (**b**) on temperature. Steam to ethanol ratio 6, ethanol inlet concentration 14 vol. %.

***S/E* = 4**

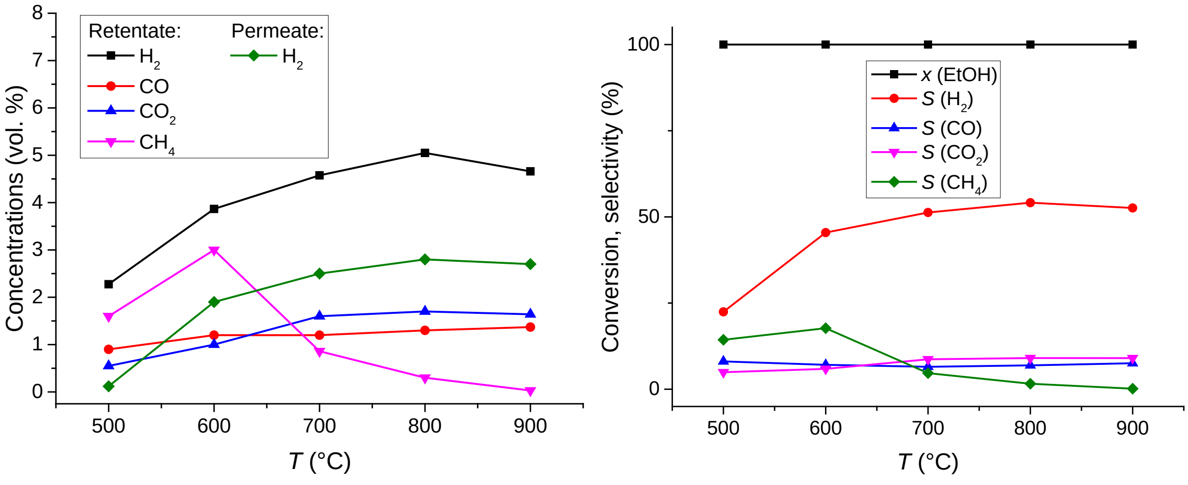

(**a**) (**b**)

Figure S6. Dependencies of ethanol steam reforming products concentrations (**a**), ethanol conversion and product selectivities (**b**) on temperature. Steam to ethanol ratio 4, ethanol inlet concentration 4 vol. %.

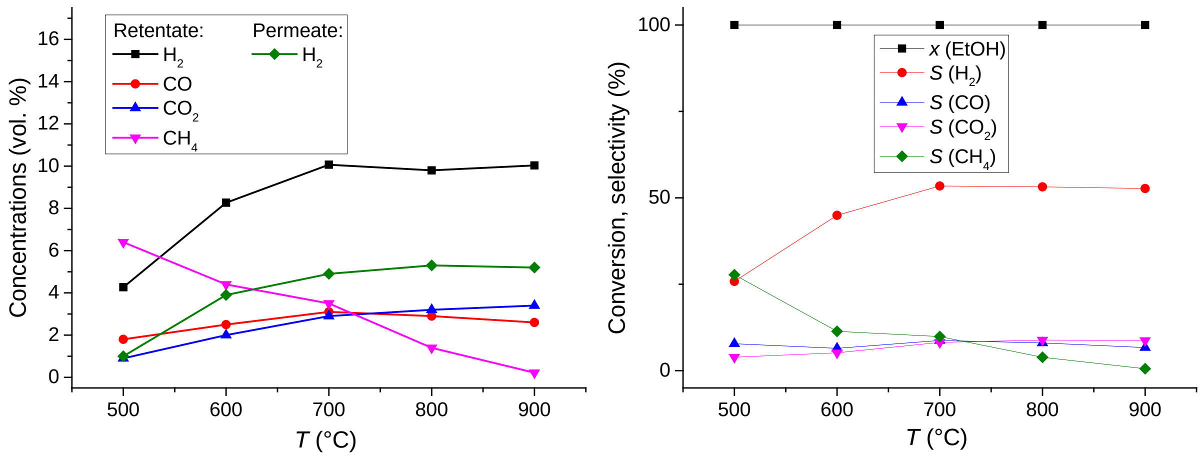

(**a**) (**b**)

Figure S7. Dependencies of ethanol steam reforming products concentrations (**a**), ethanol conversion and product selectivities (**b**) on temperature. Steam to ethanol ratio 4, ethanol inlet concentration 8 vol. %.

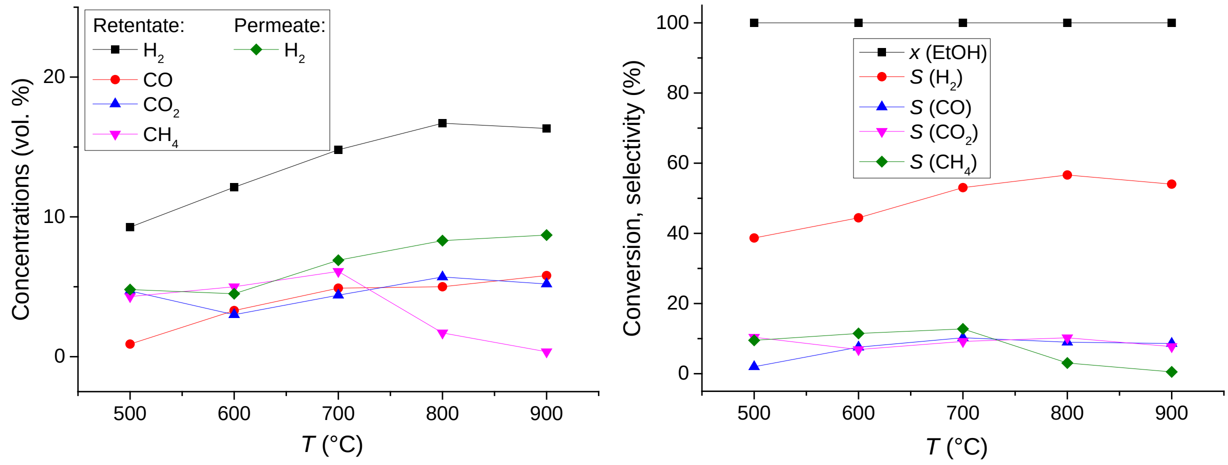

(**a**) (**b**)

Figure S8. Dependencies of ethanol steam reforming products concentrations (**a**), ethanol conversion and product selectivities (**b**) on temperature. Steam to ethanol ratio 4, ethanol inlet concentration 12 vol. %.

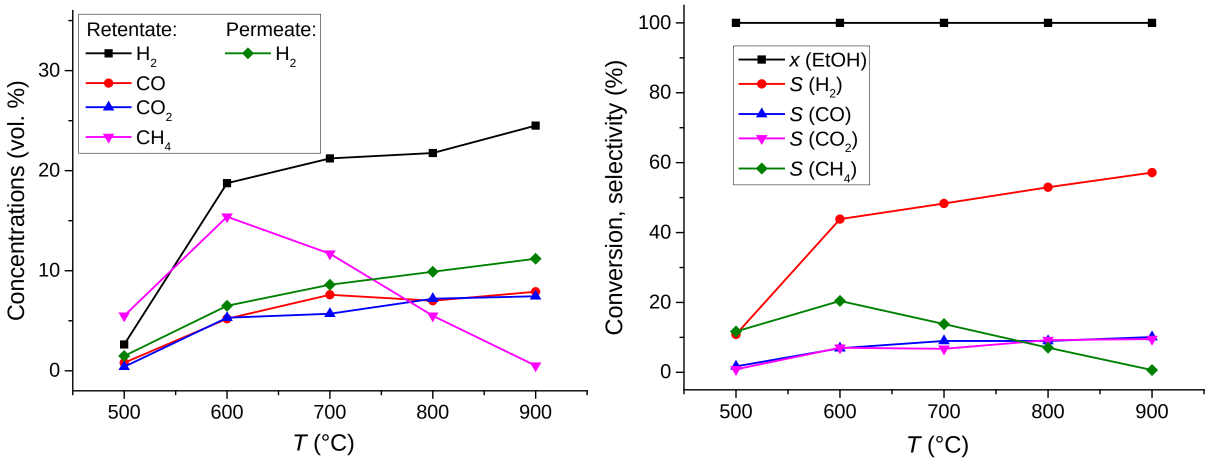

(**a**) (**b**)

Figure S9. Dependencies of ethanol steam reforming products concentrations (**a**), ethanol conversion and product selectivities (**b**) on temperature. Steam to ethanol ratio 4, ethanol inlet concentration 16 vol. %.

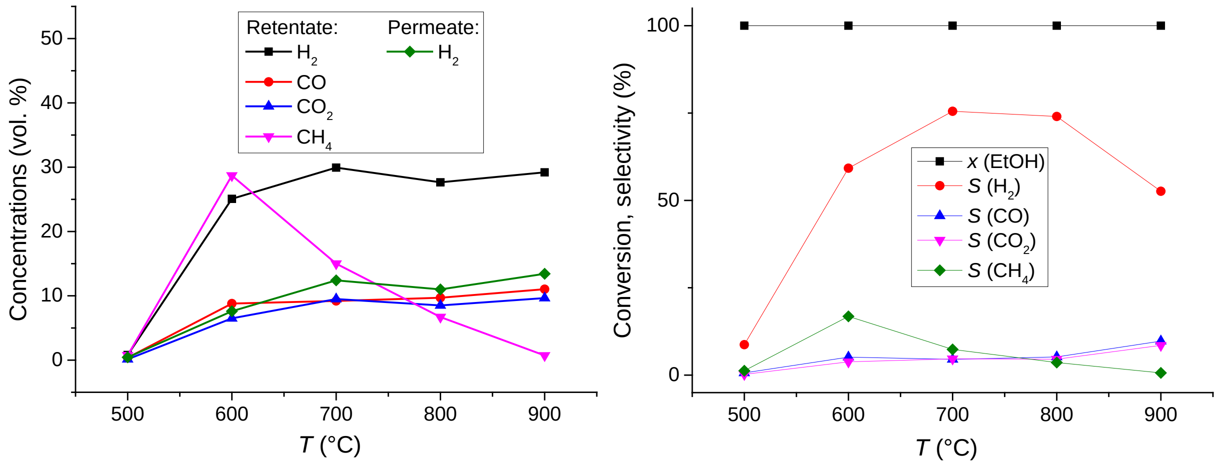

(**a**) (**b**)

Figure S10. Dependencies of ethanol steam reforming products concentrations (**a**), ethanol conversion and product selectivities (**b**) on temperature. Steam to ethanol ratio 4, ethanol inlet concentration 20 vol. %.

S/*E* = 2

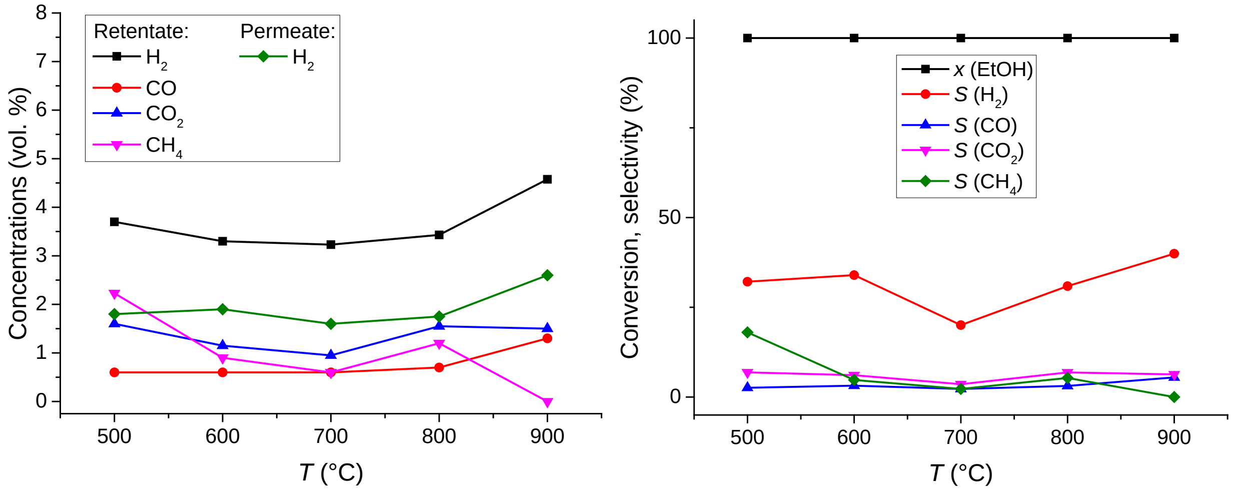

(**a**) (**b**)

Figure S11. Dependencies of ethanol steam reforming products concentrations (**a**), ethanol conversion and product selectivities (**b**) on temperature. Steam to ethanol ratio 2, ethanol inlet concentration 6 vol. %.

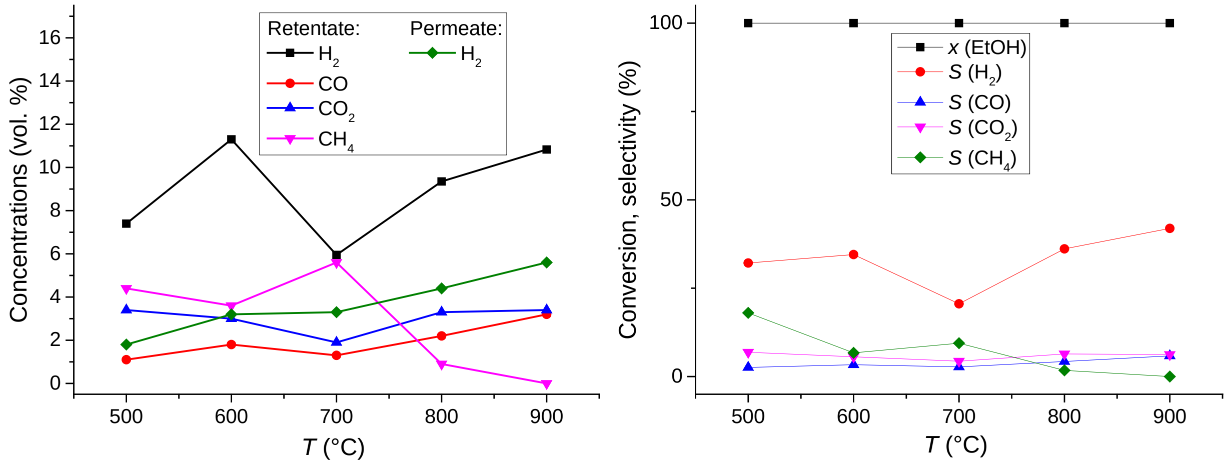

(**a**) (**b**)

Figure S12. Dependencies of ethanol steam reforming products concentrations (**a**), ethanol conversion and product selectivities (**b**) on temperature. Steam to ethanol ratio 2, ethanol inlet concentration 13 vol. %.

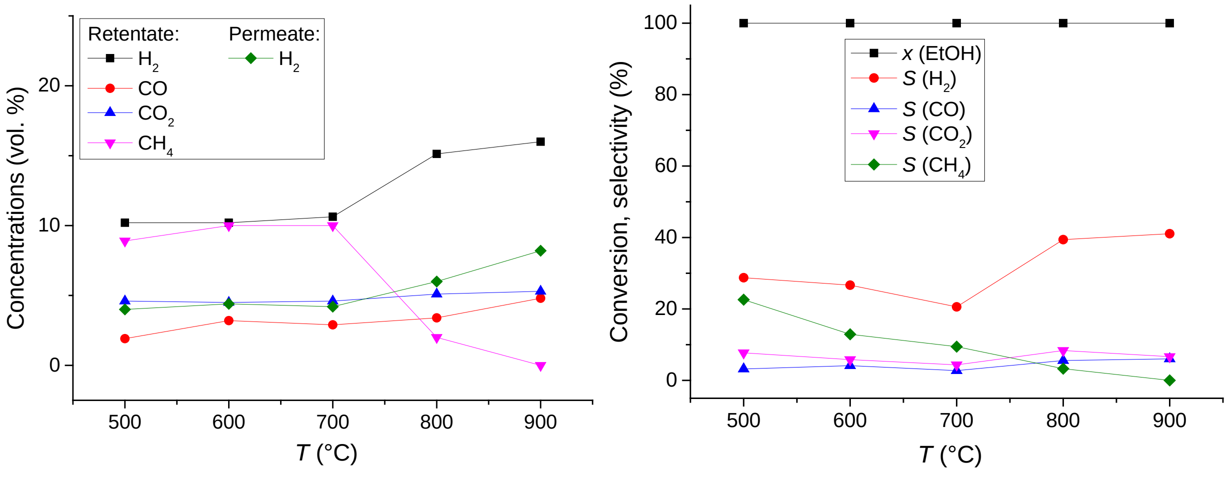

(**a**) (**b**)

Figure S13. Dependencies of ethanol steam reforming products concentrations (**a**), ethanol conversion and product selectivities (**b**) on temperature. Steam to ethanol ratio 2, ethanol inlet concentration 20 vol. %.

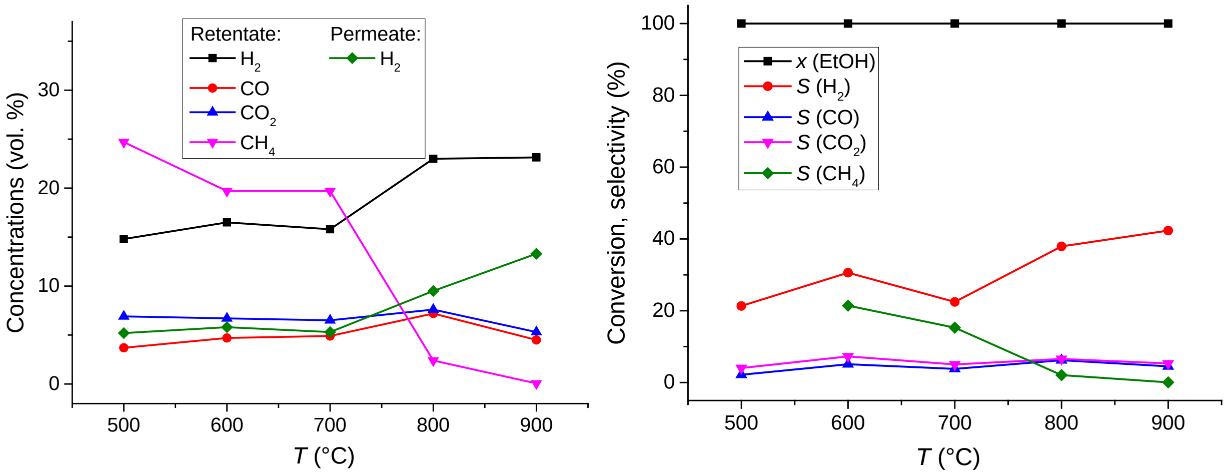

(**a**) (**b**)

Figure S14. Dependencies of ethanol steam reforming products concentrations (**a**), ethanol conversion and product selectivities (**b**) on temperature. Steam to ethanol ratio 2, ethanol inlet concentration 27 vol. %.

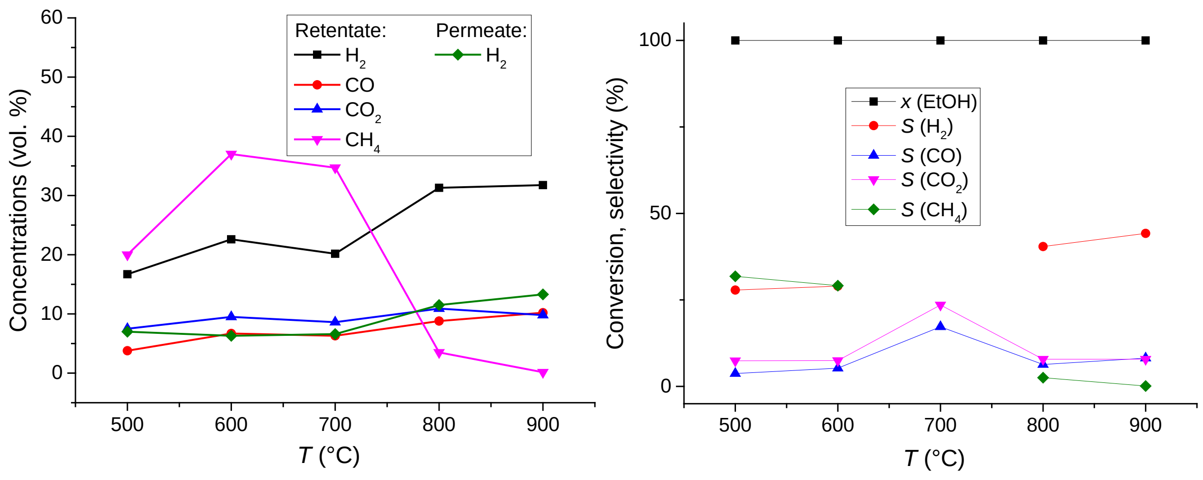

(**a**) (**b**)

Figure S15. Dependencies of ethanol steam reforming products concentrations (**a**), ethanol conversion and product selectivities (**b**) on temperature. Steam to ethanol ratio 2, ethanol inlet concentration 33 vol. %.
